# Alternative Pathways of IL-1 Activation, and Its Role in Health and Disease

**DOI:** 10.3389/fimmu.2020.613170

**Published:** 2020-12-18

**Authors:** Katerina Pyrillou, Laura C. Burzynski, Murray C. H. Clarke

**Affiliations:** Division of Cardiovascular Medicine, Department of Medicine, University of Cambridge, Addenbrooke’s Hospital, Cambridge, United Kingdom

**Keywords:** cytokines, interleukin-1, inflammasome, innate immunity, inflammation, adaptive immunity

## Abstract

Cytokines activate or inhibit immune cell behavior and are thus integral to all immune responses. IL-1α and IL-1β are powerful apical cytokines that instigate multiple downstream processes to affect both innate and adaptive immunity. Multiple studies show that IL-1β is typically activated in macrophages after inflammasome sensing of infection or danger, leading to caspase-1 processing of IL-1β and its release. However, many alternative mechanisms activate IL-1α and IL-1β in atypical cell types, and IL-1 function is also important for homeostatic processes that maintain a physiological state. This review focuses on the less studied, yet arguably more interesting biology of IL-1. We detail the production by, and effects of IL-1 on specific innate and adaptive immune cells, report how IL-1 is required for barrier function at multiple sites, and discuss how perturbation of IL-1 pathways can drive disease. Thus, although IL-1 is primarily studied for driving inflammation after release from macrophages, it is clear that it has a multifaceted role that extends far beyond this, with various unconventional effects of IL-1 vital for health. However, much is still unknown, and a detailed understanding of cell-type and context-dependent actions of IL-1 is required to truly understand this enigmatic cytokine, and safely deploy therapeutics for the betterment of human health.

## Part 1—IL-1 Is Activated by Proteolysis

**Figure 1:**
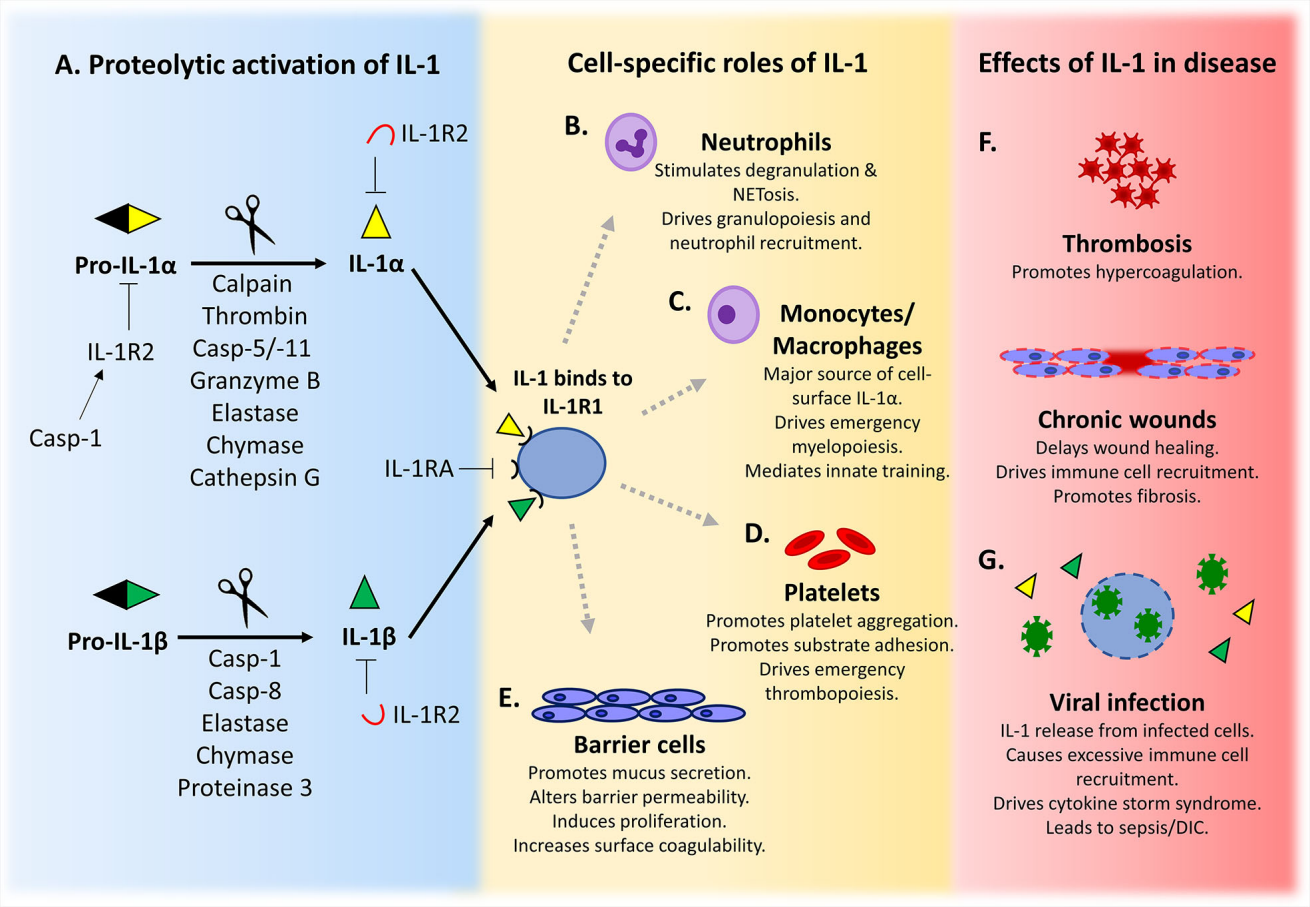
IL-1 is activated after proteolysis and has a variety of cell-specific roles that maintain homeostasis. **(A)** Pro-IL-1α/β is activated after cleavage by a diverse range of proteases. Active IL-1 binds to its signalling receptor IL-1R1 and elicits downstream signalling. IL-1 signalling is inhibited by binding to soluble or cell surface decoy receptor IL-1R2, or by competition from the IL-1 receptor antagonist (IL-1RA) for IL-1R1. **(B–E)** The production and response to IL-1 by specific cell types is important for the maintenance of cell function and homeostasis. **(F, G)** Dysregulation of IL-1 signalling can exacerbate or drive development of disease.

IL-1α and IL-1β are the most studied members of the IL-1 superfamily, and although both ligate the same receptor (IL-1R1), and therefore induce identical downstream signaling, activation pathways of the two cytokines differ. Both IL-1α and IL-1β are expressed as proforms that require proteolytic processing for maximum cytokine activity (1), with removal of the N-terminus (N-term) leading to unmasking of key residues and/or a conformational change that enables the signature C-terminal beta-trefoil motif to interact with the receptor. The study of IL-1 activation has historically focused on processing of IL-1β by caspase-1 (casp-1) (2) and IL-1α by calpain (3). However, as summarized in **Figure 1A**, pro-IL-1 processing and activation can be mediated by caspases other than casp-1 and a range of other tissue and/or cell-type specific proteases. Together, this allows IL-1 activation to be controlled in specialized environments across the body (1). This review focuses on alternative modes of IL-1 activation and the more unconventional effects IL-1 can have in health and disease. Throughout the review use of the term “IL-1” relates to effects that can be driven by either IL-1α or IL-1β, or where experiments could not determine the form responsible (e.g. use of an *Il1r1* knockout), whilst use of “IL-1α” or “IL-1β” refers to processes that were driven by a specific form.

### IL-1 Activation *via* Canonical NLRP3 Inflammasomes

Typical IL-1β activation is mediated by casp-1 cleavage after inflammasome formation. Activation of inflammasomes is extensively reviewed elsewhere (1, 4). Briefly, inflammasomes are intracellular multiprotein complexes that assemble in response to pathogen-associated molecular patterns (PAMPs) (e.g. bacterial LPS, viral dsRNA) or danger-associated molecular patterns (DAMPs) (e.g. uric acid crystals, ATP), as well as other environmental factors (5). The NOD-, LRR-, and pyrin domain-containing protein 3 (NLRP3) inflammasome is the best studied and unique in its ability to form in response to a wide range of stimuli. Canonical activation occurs after the sensing of factors by NLRP3, causing assembly of NLRP3, ASC and casp-1 into a complex that results in the activation of casp-1. Active casp-1 cleaves pro-IL-1β and pro-IL-18 to active cytokines and cleaves Gasdermin D (GSDMD) to a form able to generate pores in the plasma membrane, both releasing cytokines and inducing pyroptotic cell death by loss of plasma membrane integrity (6, 7). Furthermore, IL-1α is also able to be released from cells *via* GSDMD pores (8), but it is unclear if proteolytic processing of pro-IL-1α is required prior to release.

### Non-Canonical Activation of NLRP3 Inflammasomes

Non-canonical inflammasome activation typically occurs in response to intracellular bacteria or internalized LPS. Depending on the cell type, non-canonical activation of inflammasomes occurs *via* a one- or two-step process. Macrophages require two-steps, with an initial engagement of a pathogen recognition receptor by PAMPs (e.g. LPS binding to toll-like receptor 4 (TLR4)) leading to NF-κB-mediated upregulation of inflammasome components (e.g. NLRP3 and pro-casp-1) and type I interferon (IFN) signaling-mediated upregulation of pro-casp-5 (in humans) or casp-11 (in mice), complementing the constitutively expressed human pro-casp-4. In the second step, these non-canonical caspases bind to intracellular LPS *via* the Lipid A domain, leading to their activation (9–11) and the subsequent cleavage of GSDMD. The now active N-term of GSDMD forms pores in the plasma membrane, to drive pyroptosis, and in turn triggers NLRP3 inflammasome activation (4, 12). In the event of infection with cytosol-invading bacteria such as *Salmonella typhimurium* or *Shigella flexneri*, where the Lipid A moiety is not accessible, interferon-induced guanylate-binding proteins (GBPs) are critical for LPS recognition and activation of non-canonical caspases, due to their association with the vacuole containing the phagocytosed pathogen. The GBPs coating the vacuole either recruit the caspase to the vacuole to create a platform for LPS detection and casp-4 activation (13), or mediate vacuolar rupture that exposes LPS to the cytosol (14). GBP1 initiates the formation of the platform, followed by GBP2, GBP4 and GBP3, eventually controlling casp-4 recruitment and activation (15). In contrast, non-canonical inflammasome activation in human monocytes occurs in a single step, with internalized free LPS directly activating ready-made casp-4/5 (16), negating the need for IFN signaling.

Recent developments identified an alternative pathway of non-canonical inflammasome activation involving caspase-8 (casp-8). Casp-8 is best known for transmitting pro-apoptotic signals downstream of death receptor signaling during extrinsic apoptosis (17). However, casp-8 is also able to promote both the upregulation of pro-IL-1β and its activation by direct processing at the same site targeted by casp-1 (18, 19). This occurs in dendritic cells (DCs) exposed to fungi, with Dectin-1 signaling causing formation of a non-canonical casp-8 inflammasome that upregulates and matures IL-1β (20). Similarly, bacterial infection inside macrophages also induces casp-8-dependent IL-1β secretion (21, 22). However, as casp-8 can also mediate casp-1 activation (21, 22), it is likely that pro-IL-1β is processed by both casp-1 and casp-8. Stimulation of DCs and macrophages with cellular stressors (e.g. chemotherapeutics) also causes direct cleavage of pro-IL-1β by casp-8, and casp-8-mediated NLRP3 inflammasome activation (23). However, casp-8 can also induce IL-1β maturation completely independent of casp-1. In DCs where casp-1 proteolytic activity is inhibited by casp-1 mutation, resulting in no GSDMD activation or pyroptosis, casp-8 is recruited to the NLRP3 inflammasome, which enhances casp-8 activity and drives IL-1β release, albeit in a delayed fashion due to either reduced processing or release (24). Casp-8 can also mediate IL-1β release completely independent of the NLRP3 inflammasome after engagement of DC Fas with Fas ligand (FasL) on invariant natural killer (NK) T cells (25). Finally, casp-8 can mediate an alternative form of NLRP3-dependent cell death coined incomplete pyroptosis, which is driven by gasdermin E pores in the absence of casp-1/11 (26). Although activation of these non-canonical pathways is often accompanied by pyroptosis and IL-1α release *via* GSDMD (4) or GSDME (26) pores, the exact mechanism for IL-1α release remains unclear.

### IL-1α Activation by Proteolysis

The historic view is that IL-1α does not require proteolytic cleavage for full activity. However, these conclusions were drawn from studies that did not directly compare the activity of pro- and cleaved IL-1α (27), or used recombinant forms of IL-1α that were likely denatured during the purification process (i.e. by using HPLC) (28). Further work has shown that mature IL-1α has much higher cytokine activity than pro-IL-1α (29–31), with the calpain cleaved form having a ~50-fold higher affinity for IL-1R1 than the pro-form (30). In addition, cleavage of pro-IL-1α is regulated by its binding to a cytosolic form of the decoy receptor IL-1R2, which prevents calpain cleavage. However, after inflammasome activation casp-1 cleaves IL-1R2, which releases pro-IL-1α and allows calpain cleavage to the mature form (30).

Pro-IL-1α can also be cleaved by granzyme B (a cytotoxic T and NK cell protease), neutrophil elastase and mast-cell chymase, which confers bioactivity similar to calpain and up to a ~10-fold increase in activity over pro-IL-1α (29). This was found to be important in persistent inflammatory lung conditions such as cystic fibrosis, as patient bronchoalveolar lavage fluids could process IL-1α to a mature form. Interestingly, neutrophil elastase and mast cell chymase can also cleave IL-1β, IL-18, and IL-33 (32–34), perhaps revealing historic processes that could activate the ancestral IL-1 ligand before gene duplications formed the IL-1 family (35).

Thrombin, the key protease of coagulation, is also able to directly cleave and activate pro-IL-1α (36). Pro-IL-1α is cleaved by thrombin at a (K)PRS motif that is highly conserved across disparate mammalian species, suggesting functional importance. IL-1α cytokine activity after cleavage by either thrombin or calpain is equivalent, suggesting that removal of the N-term is critical for IL-1α activity, as is seen for IL-1β. The co-localization of tissue factor (TF) (a thrombin activator) and pro-IL-1α in the epidermis means that following injury thrombin generated during hemostasis can rapidly activate IL-1α, leading to inflammation and recruitment of immune cells that can safeguard against potential infection.

In addition to responding to intracellular LPS, casp-5 and -11 can also directly process and activate IL-1α (31). Again, pro-IL-1α cleavage occurs at an Asp residue that is highly conserved between different species, and this processing partially controls the release of mature IL-1α from macrophages after both canonical and non-canonical inflammasome activation (31). Importantly, *CASP5* expression is increased in senescent human fibroblasts, and release of IL-1α, which drives the senescence-associated secretory phenotype (SASP), and SASP factors (e.g. IL-6/8, monocyte chemoattractant protein-1) is reduced without casp-5 (31). Furthermore, in an *in vivo* model of hepatocyte senescence, reducing casp-11 in senescent cells leads to their accumulation, which is caused by a reduced SASP failing to recruit immune cells. Together this suggests that casp-5/11 plays a key role in regulating IL-1α activation and release in both myeloid and senescent cells.

All the proteases described above cleave pro-IL-1α within the same target region located between the N-term propiece and the C-term cytokine domain. Together, this means that cleavage of IL-1α by any protease results in an active cytokine that only differs by a few amino acids and has comparable biologically activity. IL-1α activation can therefore be affected in a broad range of scenarios, allowing it to act as a versatile universal danger signal.

## Part 2—IL-1 Production and Responses by Specific Cell Types

IL-1 was historically studied under many names, including leukocyte endogenous mediator, hematopoietin 1, endogenous pyrogen, lymphocyte activating factor, catabolin and osteoclast activating factor (37)—underscoring the pleiotropic effects of this widely expressed cytokine. In multi-cellular organisms cells exist in specialized niches that require distinct environmental cues to maintain homeostasis and functionality, much of which is controlled by soluble signaling factors such as cytokines and growth factors. Both IL-1α and IL-1β are active at very low concentrations, are tightly regulated and have important roles that extend beyond typical inflammation. Cell type-specific examples of both IL-1α and IL-1β production and response are discussed in this section.

### IL-1 Performs Key Roles in Controlling Innate Immune Cell Function

IL-1 signaling is vital for effective innate immunity, with most innate immune cells able to produce IL-1 and almost all mesenchymal/tissue cells able to respond to it (38). Innate responses typically occur after sensing of DAMPs or PAMPs by tissue resident immune cells (e.g. macrophages), which leads to upregulation of pro-IL-1α/β and other cytokines (e.g. TNFα). If the insult is severe enough, leaked factors such as ATP drive inflammasome activation and large-scale release of mature IL-1α/β. Importantly, as pro-IL-1α is constitutively expressed by many tissue cells, necrosis can release fully active IL-1α that may instigate a low level responses able to resolve an insult before full inflammasome activation and potential collateral tissue damage. IL-1-mediated processes important for innate responses include cytokine secretion, upregulation of adhesion, MHC and/or co-stimulatory molecules and induction of vascular leakage, ultimately leading to the recruitment, activation and instruction of immune cells (39).

#### Neutrophils

Neutrophils are the most abundant white blood cell in the circulation. They are rapidly recruited to sites of injury or infection where they phagocytose microbes and undergo degranulation to release bactericidal reactive oxygen species and proteases (40). Because of a short life span (~23–38 h), neutrophils require continuous replacement and robust mechanisms to control circulating numbers (41). Granulopoiesis and release of mature neutrophils from the bone marrow can be driven by IL-17 from Th17 cells (42), *via* granulocyte-colony stimulating factor. As Th17 differentiation is regulated by IL-1 (43) and Th17 cells produce IL-17 after exposure to IL-1 and IL-23, this gives IL-1 an indirect role in granulopoiesis (**Figure 1B**) (44, 45). More generally, infection and inflammation trigger neutrophilia *via* IL-1-induced proliferation of hematopoietic stem cells, a process known as emergency granulopoiesis (46).

IL-1 does not directly recruit neutrophils (47), rather it causes recruitment to sites of inflammation by upregulating neutrophil chemoattractants such as CXC- and CCL- chemokines in other cell types (48). For example, IL-1-dependent production of the chemokine IL-8 (CXCL8) occurs in endothelial cells (49), fibroblasts, and keratinocytes (50). IL-1 can also induce production of neutrophil chemotactic lipids including leukotriene B4 (51) and platelet activating factor (52). Recruitment of mature neutrophils to sites of infection or injury can also be driven by Th17 cell IL-17, which induces release of the neutrophil chemokines CXCL1, CXCL2, CXCL5, and CXCL8 from local endothelial and epithelial cells (53). Th17 cells also directly recruit neutrophils by production of CXCL8 (54). After administration of hydrocarbon oils to the peritoneal cavity, locally released IL-1α drives production of CXCL5 that recruits neutrophils (55).

IL-1 also drives calcium-dependent degranulation of neutrophils (56), with the exteriorized proteases (e.g. cathepsin G, elastase, and proteinase 3) subsequently able to cleave and activate multiple IL-1 family members (e.g. IL-1α, IL-1β, IL-33, IL-36) (57, 58). Indeed, it has been suggested that neutrophil proteases may be more effective at processing IL-1 family members than directly killing microbes (58), suggesting the ancestral purpose of these proteases was to activate cytokines. Proteinase 3 can also process IL-1β inside neutrophils lacking NF-κB signaling (59), which could serve as a host defense mechanism against pathogens able to evade NF-κB dependent innate immunity (60). Activated neutrophils release a meshwork of chromatin and proteases that form extracellular fibers, known as neutrophil extracellular traps (NETs), which trap and kill bacteria (61). Formation of NETs often leads to a lytic form of cell death called NETosis, but NETs can be released without cell death, known as vital NETosis (62). Interestingly, NET extrusion and NETosis are dependent on GSDMD pore formation that occurs during non-canonical inflammasome activation by intracellular LPS or bacteria (63, 64). NET formation also occurs in response to exogenous IL-1 (65, 66). In atherosclerotic plaques, cholesterol efflux-driven NLRP3 inflammasome activation in myeloid cells induces subsequent IL-1R1-dependent neutrophil accumulation and NET formation (67). Conversely, NET formation can also elicit IL-1 signaling, with the NET-associated proteases elastase and cathepsin G able to cleave and activate IL-1α (68, 69). Indeed, in diabetic wounds NET overproduction triggers macrophage NLRP3 inflammasome activation and IL-1β release, which sustains inflammation and impairs healing (70).

Finally, myelopoiesis following myocardial infarction is driven by neutrophil-derived IL-1β. Neutrophils recruited to the infarcted tissue release the alarmins S100A8 and S100A9 that bind to TLR4 on naïve neutrophils and induce NLRP3 inflammasome-dependent IL-1β secretion, which subsequently drives IL-1R1-dependent granulopoiesis in the bone marrow (71). Together, IL-1 signaling is either directly or indirectly capable of inducing neutrophil production, recruitment, degranulation, and NETosis. In turn, neutrophil proteases are able to activate IL-1 family members, which could act as a means to rapidly amplify inflammation at sites of injury or infection.

#### Macrophages

The main functions of a macrophage are to phagocytose cell debris and foreign bodies and release cytokines that orchestrate immune responses. Phagocytosis is fundamental for host defense, as it combines microbial killing with innate immune activation and the presentation of antigens to T cells. Macrophage phagosome maturation depends on progressive acidification, which activates pH-dependent proteases (e.g. cathepsins) that degrade the contents (72). Interestingly, mechanisms for macrophage cytokine release and phagocytosis overlap, with gram-positive bacteria within phagosomes inducing NLRP3 inflammasome activation. This leads to active casp-1 accumulation on phagosomes, where it controls acidification *via* the NADPH oxidase NOX2 (73, 74). To evade digestion in the phagosome, *Staphylococcus aureus* has evolved a mechanism that allows it to sequester mitochondria away from phagosomes, thereby preventing mitochondrial reactive oxygen species generation, local casp-1 activation, and subsequent phagosome acidification (75).

Macrophages can produce IL-1 in a variety of ways, as detailed above. However, macrophages appear unique in their ability to present IL-1α on the cell surface (**Figure 1C**) (76, 77). Cell surface (csIL-1α) is induced de-novo after TLR ligation and associates with the membrane *via* IL-1R2 and a glycosylphosphatidylinositol (GPI)-anchored protein, with trafficking to the membrane specifically inhibited by IFNγ (77). Because csIL-1α is the less active pro-form tethered *via* the N-term (77), it is both activated and released after cleavage, for example by thrombin (36), permitting wider ranging effects. However, because IL-33 causes macrophage activation and differentiation and IL-33 signaling *via* ST2 needs IL-1 receptor accessory protein (IL-1AcP) as a co-receptor (78, 79), this signaling complex likely sequesters IL-1AcP away from any IL-1R1 and reduces the potential for IL-1 signaling by macrophages.

Macrophages change phenotype in response to the microenvironment, typically becoming polarized to M1 (classically activated) or M2 (alternatively activated) subtypes. NLRP3 and IL-1β expression is reduced in M2 cells (80), and inhibition of NLRP3 inflammasome activation drives M2 polarization (81, 82). In keeping with this, NLRP3 inflammasome activation in *Treponema pallidum* infected macrophages induces M1 polarization and IL-1β release (83). In contrast, NLRP3 activation can also cause M2 polarization *via* upregulation of IL-4 in an inflammasome-independent process (84). Together, although NLRP3-controlled M1/M2 polarization is clearly important, the exact picture is yet to be elucidated.

#### Platelets

Platelets are key for hemostasis in response to vascular injury, but also form pathogenic thrombi. Activated platelets change shape, aggregate, degranulate, and upregulate receptors (85), but also release bioactive molecules that cause inflammation and modulate immune responses. Activation of platelets increases cell surface IL-1α (86, 87), which can be cleaved and released from the surface by thrombin (36). During stroke or brain injury, IL-1α released from activated platelets is important for driving cerebrovascular inflammation *via* activation of the brain endothelium, which enhances adhesion molecule and CXCL1 expression (**Figure 1D**) (88). Platelet microparticle-associated IL-1β also promotes platelet-neutrophil aggregation in injured lung microvasculature during sickle cell disease (89). Additionally, platelets are able to license NLRP3 inflammasome activation and IL-1 production in innate immune cells, particularly monocytes. This licensing is *via* an unknown soluble platelet factor, independent of contact, and is not IL-1 or an inflammasome component (90). Platelets also express IL-1R1, and treatment with exogenous IL-1β can activate platelets and enhance adhesion to substrates (91). Platelets can also respond to the IL-1β they release in an autocrine signaling loop after LPS stimulation (92).

Platelet count and thrombopoiesis are both tightly regulated. Normal thrombopoiesis occurs after thrombopoietin induces caspase activation in megakaryocytes, causing compartmentalized apoptosis and pro-platelet formation (93, 94). However, emergency thrombopoiesis also occurs after acute platelet loss, with IL-1α signaling causing alternative platelet production *via* megakaryocyte rupture (95). Indeed, mice with thrombin-resistant pro-IL-1α do not rapidly recover platelet count after platelet depletion, suggesting thrombin activation of IL-1α is the critical mechanism for this form of emergency thrombopoiesis (36). Platelets are at the interplay of hemostasis and inflammation and together it is clear that their role in host defense goes much deeper than their ability to cross link fibrin during hemostasis.

### IL-1 Is Vital for Adaptive Immunity

**Figure 2:**
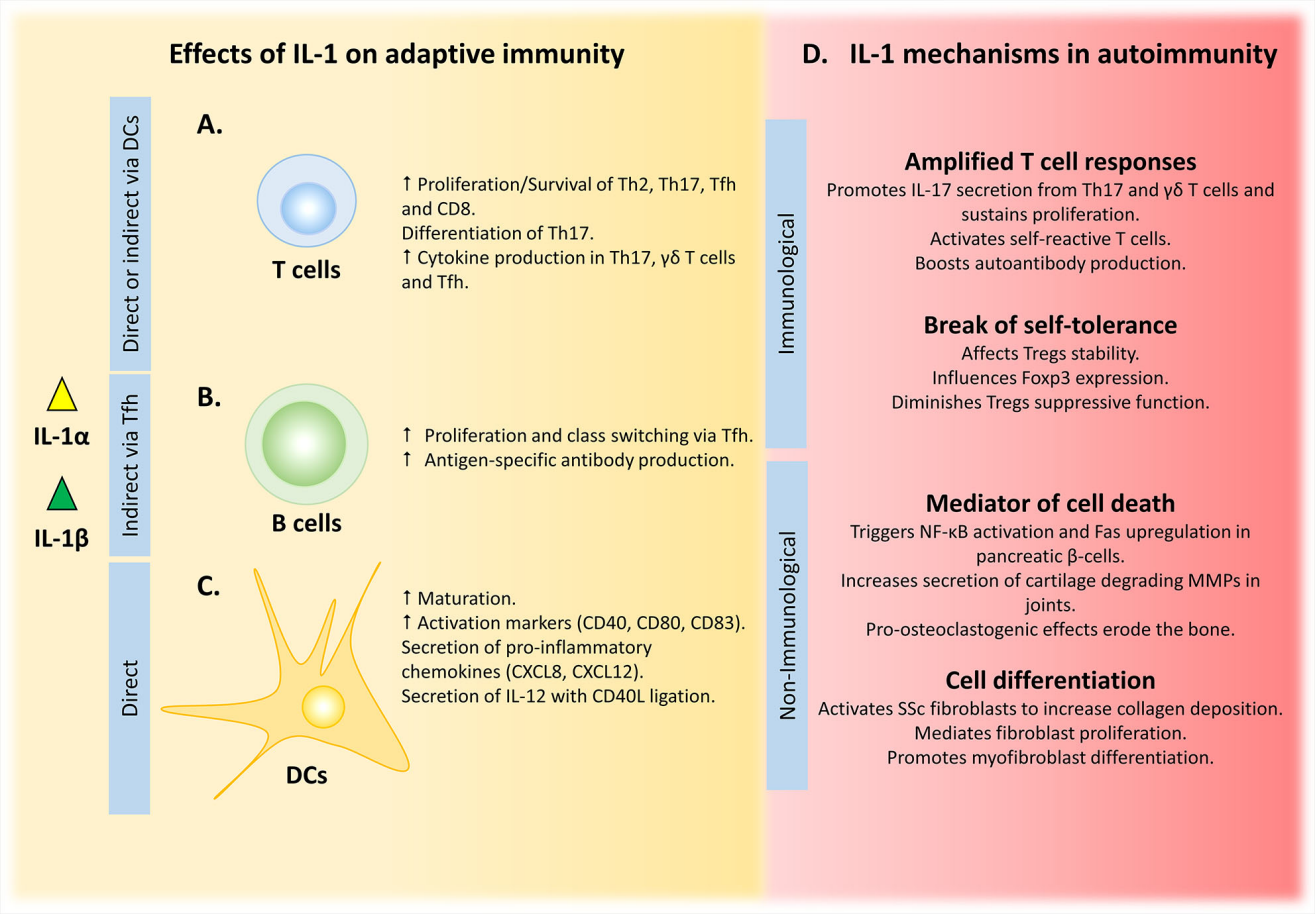
IL-1 influences adaptive immunity by multiple mechanisms, with dysregulation leading to disease. **(A)** IL-1 can shape T cell responses either by direct binding to T cell IL-1R1 or indirectly via IL-1-primed DCs. **(B)** Amplified B cell responses are driven by IL-1-activated Tfh cells. **(C)** Maturation, activation and cytokine secretion by DCs is directly driven by IL-1 signalling. **(D)** Uncontrolled IL-1 amplifies T cell responses and can break self-tolerance to drive autoimmunity, while its non-immunological role in tissue damage and remodelling can exacerbate these conditions.

An appreciation of the effects of IL-1 on the adaptive immune system dates to the 1970s, when IL-1 was originally named lymphocyte activating factor) to account for its lymphoproliferative activity (96–98). Indeed, T cell expansion under the influence of either IL-1α or IL-1β is demonstrated for a variety of T helper (Th) cell subsets such as Th1 (99, 100), Th2 (100, 101), and Th17 (100), in a process that is antigen and/or T cell receptor (TCR)-dependent. These observations are partly explained by a pro-survival role of IL-1 on the cells (100); however, other studies show no pro-survival effects of IL-1 downstream signaling on CD4 T cells (102). Similar observations were noted with Granzyme B+ CD8+ T cells, where IL-1β regulates expansion and effector function (103). IL-1α also drives Th cell differentiation, in particular Th17, but also synergizes with TNF to enable IL-12-driven Th1 differentiation (99). Th17 lineage commitment in both human (104, 105) and mouse systems (43) is conferred by IL-1-driven upregulation of the transcription factor *Rorc* (106). Production of signature cytokines by the majority of CD4 effector T cells is also augmented by other IL-1 family members (e.g. IL-18, IL-33 and IL-36), with Th9 (107, 108) and Th17 (106) cells directly responding to IL-1, which bypasses the requirement for TCR engagement and CD28 co-stimulation (100). Indeed, the tri-fold effects of IL-1 on T cell proliferation, differentiation and cytokine secretion in the absence of TCR stimulation is suggestive of the negative effects dysregulated IL-1 might have on adaptive immunity (as summarized in **Figure 2A**). This is further compounded by the inability of Tregs to effectively suppress IL-1-driven T cell proliferation and cytokine production (102). Thus, IL-1-driven activation of effector T cells with reduced Treg suppression could breach tolerance and drive autoimmunity.

#### Direct Effects of IL-1 on T Cells

It is not totally clear whether the effects of IL-1 on T cells are direct or indirect. Direct regulation implies expression of IL-1 receptor 1 (IL-1R1) on T cells, and although some evidence supports this it is not currently conclusive. Competitive binding experiments using radiolabeled IL-1 demonstrates that Th2 cells, but not Th1, bind IL-1 (109), which is supported by flow cytometry for IL-1R1 expression on murine Th2 cells (110). However, circulating human Th17 precursor cells (CCR6+ CD161+ CD4+) do not express *Il1r1* mRNA (111), and a subset of Th2 cells with the ability to express IL-17 (CRTH2+ CD4+) contain very low levels of IL-1R1 protein (112). In contrast, *bona fide* Th17 cells show clear *Il1r1* transcription (43) and expression of functional IL-1R1 on the cell surface (106). Furthermore, the presence of IL-1R1 on naive or memory CD4 T cells isolated from human blood determines Th17 fate and subsequent secretion of IL-17, with or without TCR triggering (113). Importantly, formation of an active IL-1 signaling complex requires IL-1R accessory protein (IL-1RAcP), and therefore the expression pattern of this co-receptor in conjunction with IL-1R1 is required to determine which T cells respond to IL-1 (114, 115). CD4 and CD8 T cells express cell surface IL-1RAcP, with higher abundance on regulatory CD4+ CD127^low^ CD25^hi^ cells that also express high levels of forkhead box P3 (FoxP3) (116). IL-1RAcP is also stably expressed on Th2 cells, where it acts as a co-receptor for ST2 that mediates T helper type 2 reactions in response to IL-33 (117, 118), suggesting that Th2 cells would respond to IL-1 if IL-1R1 is co-expressed.

T follicular helper (Tfh) cells also express IL-1R1 transcript and protein, and functionally respond to IL-1 (119), suggesting they must also express IL-1RAcP. Tfh cells provide growth and differentiation signals (i.e. IL-4, IL-21 and ICOS) to germinal center (GC) B cells, which drives affinity maturation, somatic hypermutation and long-lived humoral immunity (**Figure 2B**) (120–122). Indeed, administration of IL-1β during immunization substitutes for adjuvant-induced Tfh expansion and cytokine release (specifically IL-4 and IL-21). Moreover, IL-1β augments adjuvant-driven immunization by enhancing antigen-specific antibody production. In contrast, IL-1RA (Anakinra) inhibits these responses, showing the importance of IL-1 signaling in Tfh cells (119). The need of IL-1β for a productive GC and antibody response after immunization (123) suggests that driving IL-1-dependent Tfh-mediated B cell activation might be beneficial for enhancing vaccine efficacy, either by direct IL-1 administration or by using agents that induce or release IL-1 (e.g. alum and necrotic cell death, respectively). However, GC activation has to be tightly regulated, because loss of control could breach B cell tolerance and allow self-reactive clones to escape the GC, ultimately causing systemic autoimmune disease (124, 125).

Tregs maintain immune homeostasis by suppressing unwanted immune responses *via* multiple mechanisms (126, 127), including regulation of IL-1 availability *via* expression of IL-1 antagonists. Within the GC, Treg subtypes can restrain humoral responses by inhibiting secretion of the B cell activating cytokines IL-4 and IL-21 from activated Tfh (128, 129). T follicular regulatory (Tfr) cells in particular express high levels of IL-1R2 and IL-1RA, which suppresses IL-1-driven GC activation by inhibiting Tfh activation and secretion of IL-4 and IL-21 (119). Thus, in cases of IL-1-mediated GC dysregulation, IL-1 antagonists may be useful in controlling these responses and restoring protective immunity. However, non-physiological upregulation of IL-1R2 represents a maladaptive mechanism utilized by Tregs infiltrating colorectal, non-small cell lung or breast cancers that are more aggressive with poor prognosis (130, 131). Supported by evidence showing that DC-derived IL-1β-dependent priming of CD8+ T cells augments their tumoricidal properties and that *Nlrp3^-/-^* or *Casp-1^-/-^* DCs sub-optimally prime T cells (132), these data suggest that IL-1β (or potentially IL-1α) depletion from the tumor microenvironment could confer immunosuppression and poor tumor control, and thus IL-1R2 expression on intra-tumoral Tregs could be utilized as a prognostic marker.

#### Indirect Effects of IL-1 on T Cells *via* Dendritic Cell Activation

In addition to the direct effects of IL-1 on T cells, their function can also be indirectly influenced by interaction with IL-1-primed DCs (**Figure 2C**). Both IL-1α or IL-1β-pulsed DCs enhance T cell-dependent cytokine secretion from CD4 and CD8 T cells, with IFN-γ secretion being consistently augmented (133–135). Although IL-1β is not as strong an inducer of DC maturation as LPS, it still potently upregulates activation markers (e.g. CD40, CD80 and CD83) and induces secretion of pro-inflammatory mediators (e.g. CXCL8 and CXCL12) (133). In contrast, IL-1β induces minimal secretion of the major polarizing cytokine IL-12p70, but in combination with CD40L both expression of *Il12b* and release of IL-12p70 is increased (134, 136). In addition, antigen endocytosis, which is diminished during DC maturation, is decreased by IL-1β to a similar degree as LPS (137). What is clear is that the role of IL-1 on adaptive immunity is multifaceted and cannot be summarized in a single conclusion. A multitude of normal immune responses depend on IL-1 signaling, including T cell proliferation, expansion and differentiation, as well as humoral responses to T cell-dependent antigens. Moreover, IL-1 synergizes with CD40L to potentiate activation and maturation of DCs, and the subsequent adaptive immune response. Together, these processes are critical for host defense, and dysregulation of the equilibrium leads to either an ineffective adaptive reaction or an overwrought maladaptive response that drives autoimmune pathology.

### IL-1 Is Important for Maintenance of Epithelial Barriers

Epithelial cells form a highly specialized physical barrier against the external environment, and thus are exposed to a myriad of PAMPs, damage-associated molecular patterns (DAMPs), and other environmental factors that induce inflammation. Although inflammasomes are primarily studied in myeloid cells they are also essential for barrier defense in non-myeloid cells, including specialized epithelial cells. Indeed, IL-1 is constitutively expressed in many epithelial cells, where it helps defend against pathogens and injury (**Figure 1E**) (138). However, loss of control can lead to inappropriate activation, chronic inflammation and disease (139).

#### Keratinocytes

Keratinocytes secrete pro-IL-1α that can be processed by thrombin activated during hemostasis after wounding. This connection between the coagulation and immune systems allows rapid IL-1α activation and immune cell recruitment that can safeguard against potential infection after breach of the epidermal barrier (36). In response to UVB irradiation (140) or analogs of viral double stranded RNA (141) keratinocytes activate the NLRP3 inflammasome and secrete IL-1β. The IL-1 released from keratinocytes after viral infection induces expression of anti-viral interferon-stimulated genes in local fibroblasts and endothelial cells. However, this is not observed in mouse models, suggesting human skin has an alternative IL-1-driven anti-viral system to prevent viruses that can evade detection by pattern recognition receptors (142).

#### Gastrointestinal Epithelium

The gastrointestinal tract epithelium is highly specialized throughout its length and must respond appropriately to its local environment to maintain homeostasis. In the stomach, IL-1-mediated reduction of gastric acid production (143–145) and prostaglandin synthesis (146) by parietal epithelial cells inhibits neutrophil infiltration, which protects the gastric lining from non-steroidal anti-inflammatory drug-induced gastropathy (146) and ulceration (147). In the duodenum, IL-1 signaling causes epithelial goblet cells to increase mucus secretion (148, 149), and IL-1 can also confer protection to disrupted mucosal layers during *Helicobacter pylori* infection (150). In contrast, elevated IL-1 signaling can also contribute to gut epithelium dysfunction by increasing tight junction permeability (151, 152) and inhibiting effective absorption (153–155). Finally, IL-1 signaling in colonic epithelial cells plays a direct pro-tumorigenic role by regulating the early proliferation and survival of colorectal cancer cells, independent of inflammation (156).

#### Respiratory Epithelium

The respiratory epithelium comprises basal, ciliated, and secretory epithelial cells responsible for homeostatic regulation of lung fluid, clearance of inhaled agents, recruitment of immune cells, and regulation of airway smooth muscle function (157). Epithelial IL-1 signaling regulates mucin secretion and airway surface liquid metabolism, resulting in enhanced mucociliary clearance during inflammation (158). In cystic fibrosis, where CFTR-mediated fluid secretion is impaired, IL-1 drives secretion of epithelial mucin, but not fluid, causing mucus hyperconcentration (159). IL-1 and TNFα also promote the regeneration of alveolar epithelial cells by stimulating proliferation of type 2 epithelial progenitor cells (160). Indeed, IL-1 and TNFα released during influenza infection promotes repair of damaged alveoli (161).

#### Endothelial Cells

Endothelial cells are critical for blood vessel formation, coagulation, regulation of vascular tone, and inflammation (162). Heme from ruptured red blood cells can activate the NLRP3 inflammasome and cause IL-1β release from endothelial cells, and thus inflammation can occur in response to sterile hemolysis (163). Endothelial cells activated by IL-1 express TF on their surface, which initiates the coagulation cascade (69, 164, 165). IL-1 also causes endothelial cells to reduce tight junction integrity (166–168) and increase expression of adhesion molecules, such as ICAM (169) and PECAM1 (170), facilitating diapedesis of leukocytes. In contrast, IL-1 reduces transcellular diapedesis through endothelial cells (167). Finally, IL-1 also acts as a potent proangiogenic signal *via* endothelial cell-derived vascular endothelial growth factor (VEGF) (171). For example, during tumorigenesis, IL-1 from recruited myeloid cells causes endothelial cells to upregulate VEGF and other proangiogenic factors, which promotes an inflammatory microenvironment that supports tumor angiogenesis (172, 173). In these models IL-1 inhibition restricted tumor growth and angiogenesis (174). Together, IL-1 signaling is essential to many specialized epithelial cell processes and is vital for effective barrier defense; however, dysregulation can lead to excessive inflammation and tumorigenesis.

## Part 3—Alternative Roles of IL-1 in Health and Disease

Immune responses are necessary for the maintenance of homeostasis; for example, to maintain an environment that is inhospitable for invading pathogens (e.g. mucous membranes), or in contrast, training the immune system to tolerate bacteria (e.g. gut microbiota). However, balance is everything, with loss of homeostasis leading to chronic inflammation and/or inappropriate adaptive immunity, which both exacerbates existing disease and drives emergence of new pathology. IL-1 signaling is central to many immune responses, and thus blocking IL-1 therapeutically is important in a wide spectrum of diseases (175). This section focuses on some of the more unconventional effects of IL-1 in health and disease.

### IL-1 Signaling Is Important During Wounding and Thrombosis

Skin wounding occurs when the epidermal barrier is disrupted by cutting, excessive force, chemicals, or extreme temperature. Wounding triggers a series of events critical to the healing process: hemostasis, inflammation, proliferation, and remodeling (176). Initially, hemostasis is activated after vessel wall injury to allow local thrombus formation that stymies blood loss (177). Subsequent inflammation at the wound site is integral for removal of damaged tissue and recruitment of immune cells that coordinate repair (176).

Epidermal IL-1α colocalizes with TF, a potent thrombin activator. Thus, epidermal injury results in generation of active thrombin, which cleaves and activates pro-IL-1α (36). Indeed, genetically modified mice with thrombin-resistant pro-IL-1α recruit fewer neutrophils and macrophages to the granulation tissue under the wound and show delayed healing (36). Furthermore, excisional wounds in *Nlrp3* ^-/-^ and *Casp1* ^-/-^ mice contain less IL-1β and TNFα, recruit fewer neutrophils and macrophages, and also show delayed healing that can be partially rescued by addition of exogenous IL-1β (178). In keeping with this, application of the NLRP3 activator ATP accelerates wound closure in wild type mice (179)*. Staphylococcus aureus* infection of wounds upregulates keratinocyte IL-1 that drives neutrophil recruitment *via* IL-1R1 signaling (180), while NLRP3 activation is also seen after sterile burn injuries, with Nlrp3-/- mice showing reduced macrophage infiltration and impaired healing after chemical burns (181). In contrast, excessive IL-1 can lead to chronic non-resolving wounds (**Figure 1F**). For example, sustained NLRP3 activation and elevated IL-1β is found in non-healing diabetic wounds (182), with inflammasome inhibition improving healing *via* increased angiogenesis and reduced inflammatory macrophages (182, 183). Additionally, excessive neutrophil NETs cause NLRP3-dependent IL-1β release from macrophages (70), with NET digestion reducing inflammasome activation and macrophage infiltration, leading to diabetic wound healing (70). IL-1 signaling also promotes fibrosis in the wound, with IL-1 inhibition reducing scarring and improving healing in deep tissue (184) and diabetic wounds (185). Together, this shows that while IL-1 signaling in the wound is necessary for effective wound healing, it must be tightly regulated to prevent pathological chronic inflammation and/or scarring.

Hemostasis is essential to prevent injury-related blood loss; however pathological thrombosis occurs when anticoagulant mechanisms are unable to limit excessive activation of the hemostatic pathway, leading to vessel occlusion and downstream ischemia. Excessive IL-1 signaling can cause thrombosis (**Figure 1F**), with the CANTOS study finding that IL-1β inhibition with Canakinumab reduces secondary atherothrombotic events in patients with residual inflammatory risk, which was likely due to modulation of procoagulant factors and reduced leukocyte recruitment to the plaque (186). Additionally, increased NLRP3-dependent IL-1β maturation and venous thrombosis is seen in mice deficient for the vasculoprotective enzyme CD39, which is reversed by IL-1 inhibition (187). Thrombosis relies on the interplay between blood cells, plasma proteins and the vessel wall. Neutrophils drive thrombosis by producing TF and NETs, which act as scaffolds for thrombus stabilization (188, 189), which can induce coagulopathy during sepsis (190), acute respiratory distress syndrome (ARDS) (191), and coronary artery thrombosis (192). ST-elevation myocardial infarction (STEMI) patients with elevated inflammatory markers (e.g. hsCRP) have increased circulating IL-1β and NET-associated TF, while mouse models of thrombosis have reduced NET-associated TF and delayed thrombotic occlusion when IL-1β is blocked (193). Similarly, IL-1β or NLRP3 inhibition attenuates NET-associated thrombosis in mouse models of breast cancer, supporting IL-1β as the driver of this mechanism (194). Additionally, as NET-associated proteases can activate IL-1α, this can also promote IL-1R1-dependent TF expression on the endothelial cell surface (69). Macrophages can also release TF after inflammasome activation by bacteria during sepsis, with GSDMD-dependent pyroptosis and pore formation allowing externalization of TF that triggers systemic coagulation and lethality (195, 196). Overall, wounding is a complex mechanism that requires rapid, tightly controlled, hemostasis and inflammation for effective healing. IL-1 is widely expressed in the epidermis and the vessel wall and can be activated by many proteases, and is therefore integral to the wounding response.

### Innate Immune Training Can Be Mediated by IL-1β

Trained immunity refers to a response in innate immune cells after encountering a pathogen that is more adaptive-like and confers a degree of protection against secondary exposure to an un-related infection (197). Establishment of trained immunity is mostly driven by epigenetic remodeling and metabolic changes that occur after the first exposure (198, 199). Because the epigenetic imprinting is locus-specific and allows access to transcription factors, it can alter cell identity and prime specific functions. Similarly, changes in cell metabolism also establish new cell functions (199). However, cell metabolism can affect epigenetic reprogramming that further alters the cells metabolic status, and thus these processes are inter-dependent. IL-1 family agonists are implicated in trained innate immunity, with macrophage-derived IL-18 playing a non-redundant role in training NK cells’ anti-tumor activity *via* secretion of IFN-γ (200–202). Moreover, IL-1β participates in induction of trained immunity in response to Bacille Calmette-Guerin (BCG) vaccination, where increased IL-1β and TNFα from peripheral monocytes correlates with enriched histone H3K4 tri-methylation at *Il6*, *Tnf*, and *Tlr4* promoters after *in vitro* re-stimulation with *M. tuberculosis*, heat-killed *S. aureus* or *C. albicans* (203).

Epigenetic changes after training *via* cytokines can be mediated by altered metabolism that leads to accumulation of metabolic products such as glucose, lactate (204), succinate, itaconate (205), mevalonate (206) and fumarate (207). In particular, the transition from oxidative phosphorylation to aerobic glycolysis is a determining step towards trained immunity, and is associated with H3K4me3 and H3K27Ac modifications in the presence of glucose, lactate (204) and mevalonate (206). Interestingly, mevalonate accumulation in patients with hyper IgD syndrome (HIDS) that lack mevalonate kinase is associated with increased IL-1β production by LPS-stimulated monocytes (206). Similarly, glycolysis-driven accumulation of succinate in response to LPS induces macrophage IL-1β expression, which occurs *via* HIF-1α binding to the *Il1b* promoter (208).

Trained immunity is also established at a systemic/central level after exposure of bone marrow hemopoietic stem and progenitor cells (HSPCs) to pathogen-derived products, such as β-glucan, BCG, or Western diet (WD) feeding. β-glucan induces bone marrow IL-1β production that acts on HSPCs, leading to preferential myelopoiesis and metabolism that favors glycolysis and cholesterol synthesis (209). BCG vaccination also causes HSPCs to skew toward myelopoiesis and epigenetic reprogramming of macrophages, such that they upregulate *Ifng*, *Tnf*, and *Il1b* upon re-stimulation with *M. tuberculosis* (210). Indeed, BCG vaccination increases circulating IL-1β, which inversely correlates with viremia after a secondary yellow fever vaccination (211). Together, this suggests that IL-1β contributes to both training process and direct anti-mycobacterial response. Interestingly, diet can also drive trained immunity, with WD fed mice exhibiting myelopoiesis skewed towards the monocytic lineage and upregulation of genes associated with hematopoiesis, metabolism, immune cell differentiation and leukocyte activation. This WD-dependent reprogramming was long lasting (4 weeks) and was dependent on NLRP3 (212).

However, despite beneficial effects of trained immunity on host defense, the long-lasting reprogramming of innate immune cells can become maladaptive and contribute to pathology. Human monocytes trained *ex vivo* with oxidized low density lipoprotein (oxLDL) or β-glucan produce pro-atherogenic cytokines and increase foam cell formation upon re-stimulation (213). Furthermore, chronic systemic exposure to IL-1 can lead to haematopoietic stem cell exhaustion that compromises blood cell homeostasis and reduces the ability to endure replicative challenges such as transplantation (214). Under such circumstances metabolites such as itaconate, which limits aerobic glycolysis and induces anti-inflammatory and anti-oxidative programs (215–217), might be utilized to counteract innate training and re-instate immune tolerance (205).

In the brain, microglia are able to undergo priming in response to injury similar to immune training seen in macrophages in the peripheral immune system (218). IL-1 is constitutively expressed at very low levels in the normal brain but appears to have a role in some physiological processes, including regulation of sleep and synaptic plasticity (219, 220). Following neurotoxic injury, upregulation of IL-1α and IL-1β occurs rapidly in the brain (219), and facilitates recruitment of leukocytes across the blood brain barrier (221). Microglia are able to retain memory of an inflammatory state *via* histone H3 modifications, which may contribute to hyper-reactive IL-1 expression in response to subsequent inflammatory stimuli, which is seen in many neurodegenerative pathologies (218).

### Autoimmunity Can Be Driven by Aberrant IL-1 Signaling

Although IL-1 is critical for host defense, it can also contribute to autoimmune disease *via* its ability to amplify T cell responses, shift the balance for immune tolerance and its direct action on non-immune cells that induces inflammation and tissue damage (102, 222) (**Figure 2D**). Indeed, each of the above mechanisms, either alone or in combination, underlie the pathophysiology of common autoimmune diseases.

#### Amplification of T Cell Response in IL-17-Producing T Cells

IL-17-producing Th17 and γδ T cells are causal of several mouse autoimmune syndromes, such as experimental autoimmune encephalomyelitis (EAE) and collagen-induced arthritis (44, 223, 224). IL-17 production in these cell types is stimulated by IL-1, suggesting that excess IL-1 would exacerbate these conditions. Indeed, both *Il1r1^-/-^* and *Il1a/b^-/-^* mice are resistant to the development of EAE due to lower Th17 cell proliferation, less IL-17 and lower self-reactive T cell activation, leading to less autoantibodies (44, 225). Similarly, IL-1β and IL-23-treated γδ T cells drive EAE by increased secretion of IL-17 directly into the brain milieu and by indirect augmentation of IL-17 production by αβ T cells (223).

As demonstrated in the Deficiency of Interleukin-1 Receptor antagonist (DIRA) syndrome, IL-1 antagonism is critical for the amelioration of IL-17-driven diseases. DIRA is an autoinflammatory condition associated with hyperactivation of IL-1 signaling due to loss-of-function mutations in IL-1RA, with severe manifestations in skin and bone. As such, DIRA is very responsive to IL-1 blockade by administration of recombinant IL-1RA (Anakinra) (226). Furthermore, IL-1 antagonists (i.e. IL-1RA and SIGIRR) delay onset and reduce severity of mouse EAE (225, 227). Interestingly, classic drugs used to manage multiple sclerosis (MS) (i.e. IFNβ) and its clinical relapse (i.e. steroids) associate with increased systemic levels of IL-1RA and IL-1R2 (228–230). In addition, monitoring the IL-1 to IL-1RA ratio in cerebrospinal fluid and/or lesions in MS patients may predict susceptibility of relapse-onset MS, hinting towards the use of IL-1 as a biomarker (231).

In keeping, loss of NLRP3 inflammasome components (i.e. NLRP3, ASC, caspases-1 or -11) confers some protection from EAE (232–235). However, inflammasome-independent IL-1β production has also been demonstrated as a result of trans-cellular interactions between effector CD4 T cells and DCs. Thus, CD4 T cell-derived TNF induces DC expression of pro-IL-1β, which is subsequently activated by casp-8 after T cell FasL ligation of DC Fas, leading to IL-1β release and induction of EAE (236).

#### Stability of Tregs and Break of Self-Tolerance

Tregs are critical for maintenance of peripheral tolerance through their ability to suppress inappropriate activation of effector T cells. Foxp3 is essential for the suppressive functions of Tregs, and thus its expression maintains physiological T cell responses. Foxp3 expression can be reduced in highly inflammatory tissue microenvironments, especially in the presence of IL-1 and IL-6, which can convert Tregs into effector T cells that produce IFN-γ and IL-17 (237–239). Indeed, this confirms observations that IL-1β reduces susceptibility of CD4 effector/memory T cells to Treg suppression due to expansion of IFN-γ producing effector CD25+ cells (240). This is more pronounced in the autoimmune-prone NOD mice, where splenocyte IL-1β production is enhanced and IL-1β neutralization restores Treg suppression and normalizes IFN-γ secretion, altogether suggesting that breach of tolerance can be driven by IL-1β (240).

### The Action of IL-1 on Non-Immune Cells Contributes to Autoimmunity

Besides its direct immunomodulatory effects, IL-1 can also act on non-immune cells to either alter their function or direct them towards apoptosis, thus contributing to the progression of several autoimmune and metabolic syndromes. Tissue damaging effects of IL-1 have been identified in the development of Type 1 and Type 2 Diabetes Mellitus (T1/T2DM) as well as rheumatoid arthritis (RA), while in systemic sclerosis (SSc) IL-1 has been shown to mediate pathological tissue remodeling.

#### IL-1-Mediated β-Cell Apoptosis in Metabolic Syndromes

Pancreatic inflammation and β-cell deregulation and loss are determining factors in the pathogenesis of both T1/T2DM (241, 242), and IL-1 can interfere with all these processes. Pancreatic β-cells express high levels or IL-1R1 and respond to IL-1 by upregulating inflammatory cytokines and chemokines (243, 244), which in turn recruit macrophages (245) and T cells (246). In addition, IL-1β has direct cytotoxic effects on β-cells (247) *via* canonical NF-κB activation and upregulation of Fas, in response to glucose, ultimately contributing to insulin resistance and development of T2DM (248). This IL-1β-mediated glucotoxicity is also relevant to T1DM, where islet resident macrophages secrete IL-1β upon stimulation with LPS and TNF (249), leading to β-cell production of cytotoxic molecules (e.g. inducible nitric oxide synthase) (250). In addition, β-cell apoptosis can also be triggered by IL-1β and IFN-γ activation of the non-canonical NF-κB pathway (251). Indeed, glucotoxicity and cell loss is abrogated by IL-1RA, improving β-cell secretory function and glycemic control (248, 252).

#### IL-1-Dependent Degradation of Bone and Cartilage in Rheumatoid Arthritis

RA is characterized by progressive destruction of the articular joints due to IL-1β-dependent degradation of both bone and cartilage (253). BALB/c mice lacking IL-1RA spontaneously develop an inflammatory arthropathy that shares features with human RA, such as inflammatory cell infiltrates, fibrin clots, bone erosion, increased IL-1β in the affected joints and autoantibodies in serum (254). IL-1 signaling in synovial cells and chondrocytes causes upregulation and secretion of matrix metalloproteinases that degrade cartilage (255–257). In addition, IL-1 and TNF, when in the presence of T cell-derived RANKL, exert pro-osteoclastogenic effects that result in bone erosion and further joint damage (258), suggesting that IL-1 could be a druggable target for ameliorating symptoms and impeding disease progression. Indeed, neutralization of IL-1 with antibodies or natural antagonists (i.e. IL-1RA or IL-1R2) diminish local inflammation and protect the joint from bone erosion, which led to the approval of Anakinra (IL-1RA) as an effective therapeutic for management of RA (259–261).

#### IL-1 Regulates Fibroblast Differentiation in Systemic Sclerosis

SSc is an idiopathic autoimmune syndrome that exhibits fibrosis in the skin and other organs like the heart and lungs, due to fibroblast activation and deposition of extracellular matrix (262). IL-1α and IL-1β regulate IL-6 and PDGF-A expression on SSc fibroblasts, promoting collagen deposition and proliferation (263, 264) and differentiation into myofibroblasts (265–267). In addition, inflammasome activation in fibroblasts leading to IL-1β production acts in an autocrine manner to trigger expression of mIR-155, which in turn regulates collagen deposition in SSc (268–270).

### IL-1 Signaling May Be Important in the Pathogenesis of COVID-19

The novel coronavirus SARS-CoV-2 is the causative agent of the acute respiratory disease COVID-19, which has caused a global pandemic (271). Symptoms of COVID-19 range from mild to severe, with viral pneumonia leading to ARDS, sepsis/disseminated intravascular coagulation (DIC) and/or multi-organ failure identified as the major causes of death (272). A key stage of the virus lifecycle is release of newly replicated virons (273), which escape *via* the host cell’s secretory pathways or by cell lysis (274). Egress of coronaviruses depends on the coronavirus envelope (E) protein, which acts as a viroporin that forms pores in the plasma membrane, causing lysis and subsequent release of DAMPs (275–277). For example, respiratory syncytial virus-infected cells release IL-1α that activates neighboring cells to promote leukocyte recruitment (278) and interferon-mediated anti-viral mechanisms (**Figure 1G**) (142). However, excessive cell lysis and DAMP release can trigger an over-the-top innate immune response and overt production of cytokines, known as a cytokine storm, which can spill over into the circulation and cause sepsis (279). Severe SARS-CoV-2 infection causes a systemic cytokine storm and ARDS, with accompanying thrombosis (271, 280, 281). IL-1 inhibition improves pathologies associated with cytokine storms, including sepsis and DIC (282–284), and thus maybe beneficial for treating COVID-19. Indeed, Anakinra (IL-1RA) appears to dampen markers of systemic inflammation and improve ARDS in COVID-19 patients. Importantly, Anakinra has a short half-life (3h) that allows rapid discontinuation of treatment if needed, in contrast to Canakinumab (half-life 26 d) (284, 285). At the time of writing there were 15 registered clinical trials examining IL-1 blockade by Anakinra for COVID-19. However, patient selection, dosing, and outcome measures are not harmonized between studies (286), and only 11 were randomized control trials. Finally, the multi-center RECOVERY trial has recently shown that the corticosteroid dexamethasone reduces deaths by one-third in ventilated COVID-19 patients (287), reinforcing the use of anti-inflammatory agents. Interestingly, dexamethasone is long known to profoundly inhibit IL-1 production (288, 289), again signifying a likely role for this apical cytokine in human health and disease. Together, there is existing evidence that blockade of inflammatory pathways, including IL-1, is beneficial for reducing symptoms of cytokine storm in COVID-19. Further clinical trials will be essential to determine the full extent of this benefit.

## Author Contributions

KP and LB contributed equally. KP, LB, and MC conceived and wrote the review. All authors contributed to the article and approved the submitted version.

## Funding

This work was funded by British Heart Foundation Grants FS/09/005/26845, FS/13/3/30038, FS/18/19/33371, and RG/16/8/32388 to MC, the BHF Cambridge Centre for Research Excellence RE/13/6/30180, and the Cambridge NIHR Biomedical Research Centre.

## Conflict of Interest

The authors declare that the research was conducted in the absence of any commercial or financial relationships that could be construed as a potential conflict of interest.
